# Pancreatic Arteriovenous Malformations: A Case From Saudi Arabia

**DOI:** 10.7759/cureus.55340

**Published:** 2024-03-01

**Authors:** Ohud T Alharbi, Mayar Alhuqaili, Daliyah Alotaibi, Jolan S Alsaud, Rayan Almohaimeed, Abdulaziz Alomair

**Affiliations:** 1 Neurological Surgery, King Fahad Medical City, Riyadh, SAU; 2 College of Medicine, Qassim University, Qassim, SAU; 3 General Surgery, Buraidah Central Hospital, Qassim, SAU; 4 General Surgery, King Fahad Specialist Hospital, Qassim, SAU

**Keywords:** arteriovenous malformations, avm, pancreas, pancreatic arteriovenous malformation, pavm

## Abstract

Arteriovenous malformation (AVM) of the gastrointestinal tract is a rare anomaly, mostly due to congenital reasons. Patients with pancreatic AVM can live without experiencing symptoms. It can present with gastrointestinal bleeding or portal hypertension, and diagnosis can be made by computed tomography (CT) or angiography. CT findings include multiple discrete intrapancreatic vessels.

A 48-year-old man complained of abdominal pain with a sensation of fullness that radiated to the back for a month, associated with shortness of breath, loss of appetite, and unintentional weight loss of 33% in one month without nausea or vomiting. On physical examination, the abdomen was soft and lax with epigastric tenderness and a negative Murphy sign. Laboratory investigations showed high amylase with normal liver and kidney functions. CT showed pancreatic AVM. He underwent partial pancreatectomy and splenectomy. After the surgery, the patient reported an improvement in symptoms. All follow-up visits were uneventful.

Pancreatic AVM is a rare disease, and the most significant chief complaint of most patients is gastrointestinal tract bleeding. It requires imaging depending on the signs and symptoms. The primary imaging modality is CT, with subtraction angiography for confirmation. Surgical treatment is the standard of management for most patients when tolerable. Additionally, early detection of these rare anomalies can avoid massive gastrointestinal tract bleeding and the development of resistance portal hypertension and can save patients’ lives if bleeding occurs.

## Introduction

Pancreatic arteriovenous malformation (AVM) of the gastrointestinal tract is a rare anomaly, mostly due to congenital reasons. Patients with pancreatic AVM can live without experiencing symptoms. However, it can present with gastrointestinal tract bleeding such as hematemesis, melena, duodenal ulcer, and portal hypertension. Diagnosis can be confirmed by imaging techniques such as computed tomography (CT) or angiography. Imaging findings include multiple discrete intrapancreatic vessels [1]. Early detection of these rare anomalies can avoid massive gastrointestinal tract bleeding and the development of resistant portal hypertension, saving patients’ lives. Angiography is critical in the treatment plan for AVM of the pancreas. AVMs have multiple differential diagnoses, such as pancreatic neuroendocrine tumors and hypervascularity metastatic tumors [1]. Here, we report the case of a 48-year-old man with pancreatic AVM, which is a rare location for gastrointestinal tract AVM.

## Case presentation

In September 2021, a 48-year-old man presented at King Fahad Specialist Hospital in Qassim region, Saudi Arabia, with vague abdominal pain and sensation of fullness that radiated to the upper back for a month, associated with shortness of breath, loss of appetite, and unintentional weight loss from 83 kg to 55 kg (estimated at 33% loss) in fewer than six months without nausea or vomiting. This was due to the severe abdominal pain that led to an inability to tolerate oral intake and loss of appetite. He was hypertensive on atenolol, not alcoholic, with no past surgical history, abdominal trauma, or pancreatitis.

**Figure 1:**
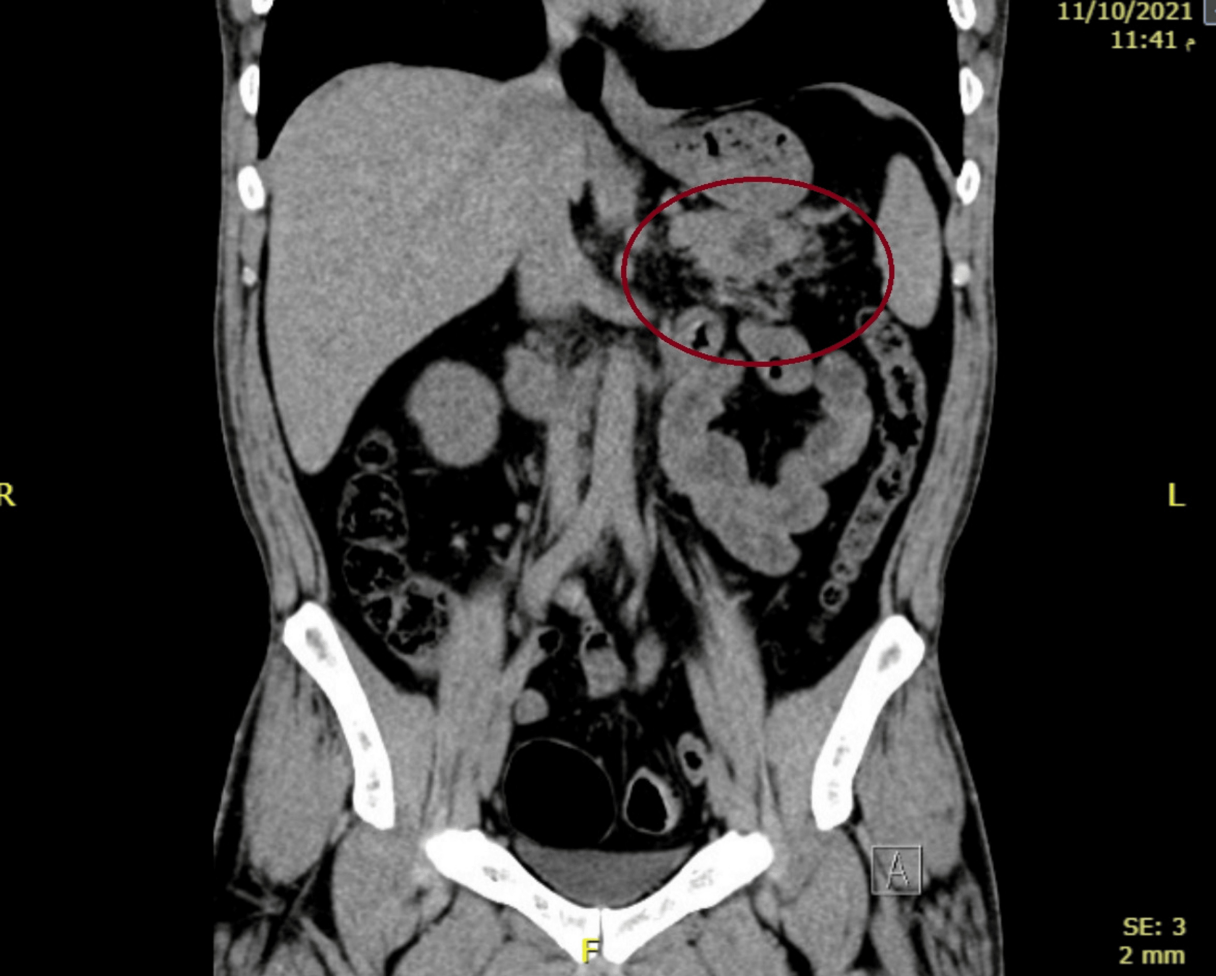
An unenhanced CT scan of the abdomen and pelvis revealing a hypodense lesion seen at the pancreatic tail associated with mild peripancreatic fatty stranding.

**Figure 2:**
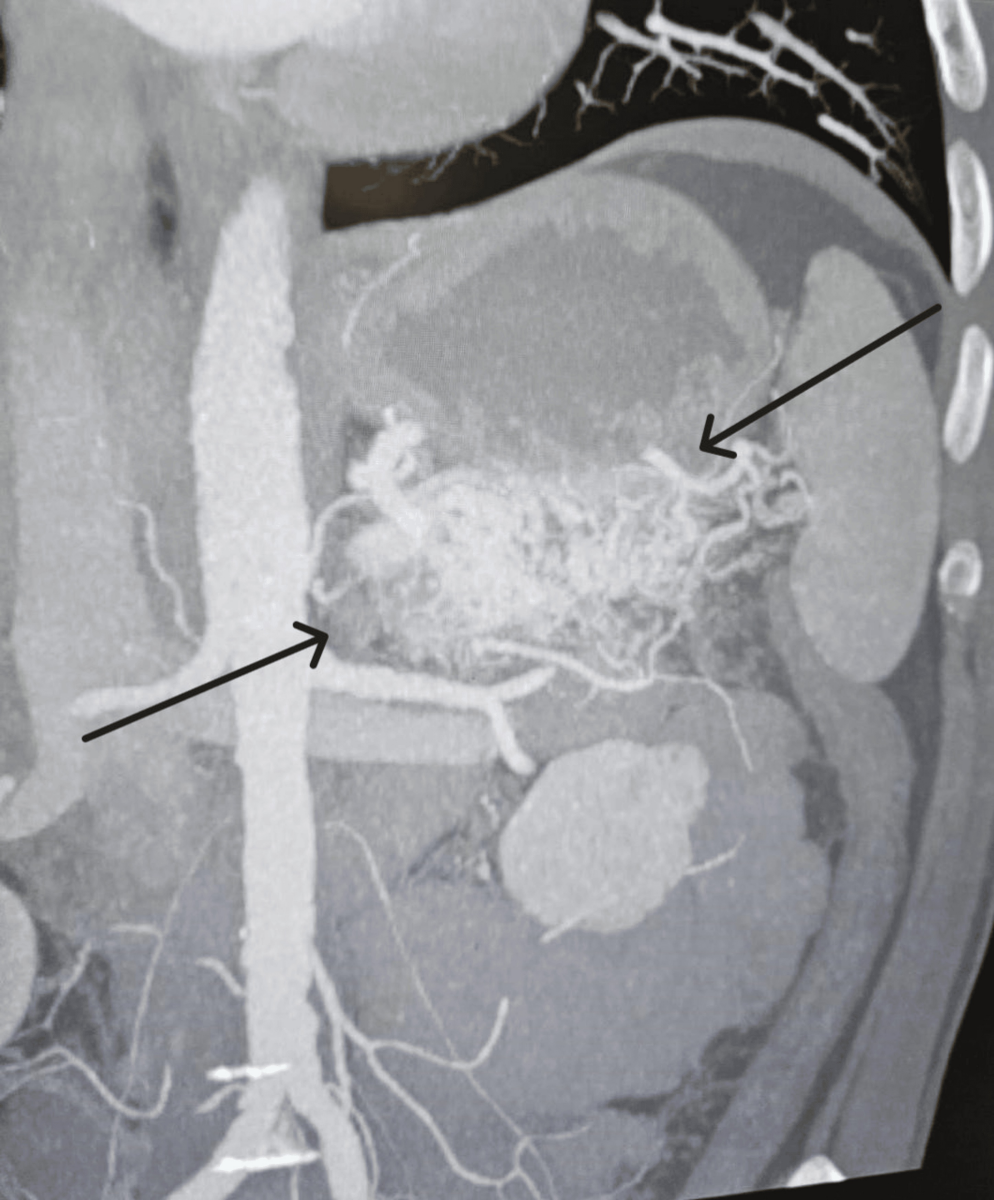
A CT scan of the abdomen and pelvis with contrast showing a pancreatic tail arteriovenous malformation.

On physical examination, the abdomen was soft and lax, with epigastric tenderness and a negative Murphy sign. Laboratory investigation showed normal random blood glucose, liver enzymes, and kidney functions with high amylase (155 U/L). An unenhanced CT scan of the abdomen and pelvis revealed a pancreatic tail hypodense lesion measuring 17 × 14 mm associated with peripancreatic fatty stranding around the tail of the pancreas (Figure 1). A CT scan of the abdomen and pelvis with contrast showed a well-defined, hypodense, non-enhancing cystic lesion at the pancreatic tail measuring about 17 × 14 mm, surrounded by a cluster of dilated collateral vessels seen in the arterial phase. Its origin was probably from the inferior pancreaticoduodenal artery which is a branch of the superior mesenteric artery and draining into the splenic vein, in keeping with AVM (Figure 2). The patient did not undergo any upper gastrointestinal endoscopy.

**Figure 3:**
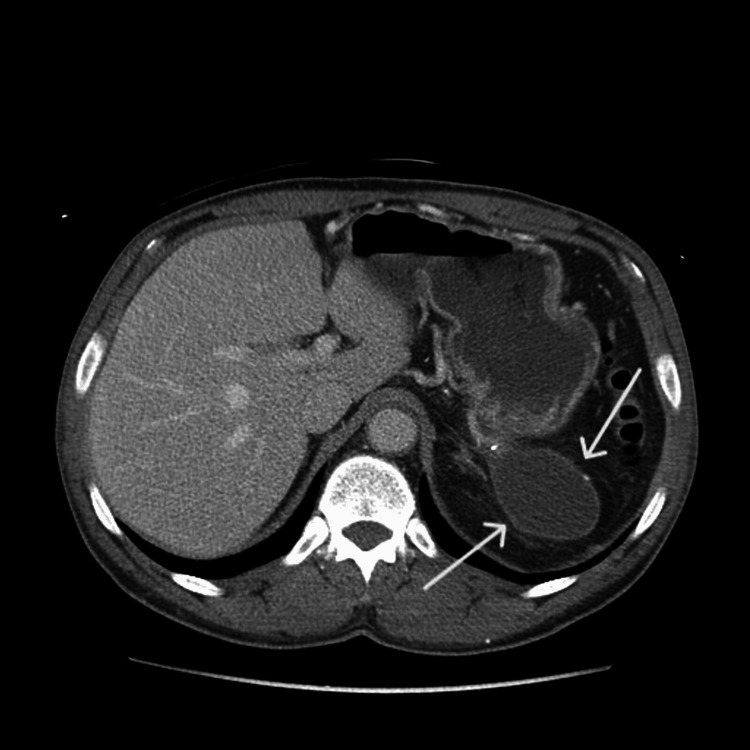
Abdominal CT with intravenous contrast showing a pseudocyst.

Intraoperative findings revealed that the malformation was located at the tail of the pancreas and adhered to the omentum dilated blood vessels and hard pancreatic tissue in that area. Partial distal pancreatectomy and splenectomy were performed. The postoperative course was uneventful, and there were no relapses during six months of follow-up. After two years, the patient complained of left loin pain, and an abdominal CT with intravenous (IV) contrast showed a pseudocyst (Figure 3). The treatment was conservative, with IV fluid and analgesics for two days, and there were no further complaints.

## Discussion

AVM is infrequent and can occur anywhere in the human body, with the pancreas being a rare location. Of all gastrointestinal tract vascular malformations, 0.9% are in the pancreas. Pancreatic AVM is a vascular anomaly with an abnormal anastomosis of the arterial and portal networks within the pancreas. Pancreatic AVM is mostly found at the pancreatic head, followed by the pancreatic body and tail [2]. Various theories have been proposed to account for the genesis of AVMs. According to Lande et al. [3], loss of regulation of the sphincter mechanism at the arteriolar-capillary junction results in an overflow of blood into the capillaries and venules, eventually forming an arteriovenous shunt. AVM in the pancreas is either congenital or acquired. Congenital vascular malformation is caused by the abnormal development of the arteriovenous plexus in the embryo, whereas pancreatitis, tumors, or trauma are causes of acquired vascular malformation. A persistent remnant of the fetal pancreatic vascular network or hereditary hemorrhagic telangiectasia, which is known as the Osler-Weber-Rendu syndrome, leads to gastrointestinal tract vascular malformation sporadically. The splenic artery, gastroduodenal, and small pancreatic arteries are the most commonly affected in pancreatic AVM. The most common presenting symptoms are gastrointestinal bleeding and epigastric pain. The bleeding has the following types of mechanisms: (1) duodenal mucosa ischemic injury by local infarction causes duodenal ulcer bleeding, resulting in abnormal vessels of pancreatic AVM; (2) The pancreatic duct or the bile duct bleeding through the orifice of the ampulla of Vater; (3) gastrointestinal tract eroded vessels bleeding by pancreatic AVM; and (4) gastroesophageal varices bleeding due to portal hypertension from pancreatic AVM.

Furthermore, the most commonly associated complications of pancreatic AVM are bleeding (50.6%), pancreatitis (16.9%), and portal hypertension [2]. Our case was unique because the pancreatic AVM presented as acute vague abdominal pain radiating to the upper back without a family history of Osler-Weber-Rendu disease or refractory duodenal ulcer. Additionally, portal hypertension was not suspected owing to the imaging findings before and after the operation. The symptoms of pancreatic AVMs are not specific, so diagnosis is usually confirmed by imaging. The incidence of this entity is very low. Angiography helps diagnose pancreatic AVM, with findings characterized by dilated and tortuous feeding arteries, a racemose intrapancreatic vascular network, early disappearance of the pancreatic stain, and early filling of the portal vein in the arterial phase itself. Magnetic resonance imaging (MRI), CT, and color Doppler ultrasonography have made diagnosing pancreatic AVM safer and more accurate. Although the waves in mosaic lesions are often flat, Doppler ultrasonography can show that the lesions are formed of pulsatile waves [3]. Esophagogastroduodenoscopy (EGD) may be normal so gastric AVM should not be ruled out even if the EGD is negative [4]. The patient in our case was accurately diagnosed with an AVM of the pancreas using contrast-enhanced CT without angiography. Surgical resection and transarterial embolization (TAE) are the primary options for treating pancreatic AVM [5]. Selective cases considered TAE as less invasive than surgical resection [6]. However, the difficulty of reducing portal hypertension, even if the pancreatic AVM is surgically removed, made the treatment with arterial embolization not preferred as it holds the risk of growing new collateral vessels, which leads to portal hypertension, recurrent gastrointestinal bleeding, and, eventually, esophageal varices rupture; another reason is that multiple feeding vessels may need to be embolized. Therefore, the definitive treatment is surgical resection of the affected lesion early to eliminate the cause of pain and prevent complications. Surgical resection should be considered before portal hypertension develops because portal hypertension is irreversible even after treatment [7]. Arterial embolization, irradiation, and transjugular intrahepatic portosystemic shunt are alternative options in patients at high surgical risk [8]. However, reducing collateral blood flow by preoperative TAE and suppressing bleeding by applying radical antegrade modular pancreatosplenectomy has been reported as useful for safe laparoscopic surgery [2].

## Conclusions

Pancreatic AVM is a rare disease that can be either congenital or acquired. The most significant chief complaint for most patients is gastrointestinal tract bleeding. The necessity of imaging depends on the signs and symptoms, with CT being the primary modality and subtraction angiography used for confirmation. Other modalities, such as MRI and Doppler ultrasonography, make the diagnosis safer and more accurate. Surgical treatment is the standard management for most patients when tolerable. Alternative options, such as irradiation, are considered in patients at high surgical risk. The treatment with arterial embolization carries the risk of serious complications. Additionally, early detection of these rare anomalies can avoid massive gastrointestinal tract bleeding and the development of resistant portal hypertension, saving patients’ lives if bleeding occurs.
